# A Multi-Centre Non-Interventional Study to Assess the Tolerability and Effectiveness of Extended-Release Tacrolimus (LCPT) in De Novo Liver Transplant Patients

**DOI:** 10.3390/jcm12072537

**Published:** 2023-03-28

**Authors:** Thomas Soliman, Georg Gyoeri, Andreas Salat, Vladimír Mejzlík, Gabriela Berlakovich

**Affiliations:** 1 Department of Surgery, Division of Transplantation, Medical University of Vienna, Waehringer Guertel 18-20, 1090 Vienna, Austria; 2 Center for Cardiovascular and Transplantation Surgery, 602 00 Brno, Czech Republic

**Keywords:** extended-release tacrolimus, real-world evidence, liver transplant, trough level, safety, liver function, graft rejection, LCPT

## Abstract

Background: Available tacrolimus formulations exhibit substantial inter- and intra-individual variability in absorption and metabolism. The present non-interventional cohort study aimed to assess the tolerability and effectiveness of the once-daily tacrolimus formulation, LCPT, in hepatic allograft recipients in real life. Materials and methods: This study was conducted in Austria and the Czech Republic between July 2016 and August 2019. Patients aged ≥ 18 years old received LCPT per the approved label and local clinical routine. All the participants provided informed consent. Patients newly treated with tacrolimus (de novo) directly after transplantation were observed for six months. The relevant clinical variables were tacrolimus trough level (TL), total daily dose (TDD), number of dose adjustments, kidney and liver function, and tolerability. Results: Of the 70 analyzed patients, 72.9% were male and 85.7% were aged < 65 years old. The mean (SD) time to achieve tacrolimus target TL was 6.4 (4.6) days after 4.4 (4.0) dose adjustments; thereafter, TL remained stable throughout observation at approximately 8 ng/mL. The LCPT TDD at initiation was 8 mg and decreased by a median of 41.4% to 5 mg at 6 months. Liver function continuously improved, and kidney function remained stable. LCPT was well tolerated with 24 adverse events in eight patients (17 related to immunosuppression, mostly mild renal insufficiency, and hematological adverse events); two serious unrelated adverse events were reported (atrial flutter and liver dysfunction). Conclusions: TL was rapidly attained with few dose adaptations after LCPT initiation in de novo liver transplant patients. Liver function rapidly improved, whereas kidney function remained normal. LCPT was well-tolerated in this population.

## 1. Introduction

The administration of immunosuppressants to transplant recipients can have two distinct objectives: prevention or treatment of allograft rejection. Although the treatment is similar in most centers, the initial immunosuppression administered to all transplant recipients may vary but typically consists of a drug combination based on a calcineurin inhibitor (CNI); steroids, with or without an antiproliferative agent; and induction therapy [1,2,3].

Tacrolimus binds to an immunophilin [4] that inhibits the activity of calcineurin and thus blocks T-cell activation and proliferation. The suppression of T-cell activation subsequently inhibits the generation of cytotoxic lymphocytes, thus downregulating processes leading to acute graft rejection [5]. However, tacrolimus concentrations may be influenced by many factors. Hence, the pharmacokinetic profile of tacrolimus shows high inter- and intra-individual variability, and the actual drug exposure does not directly correlate with the administered dose [6].

In clinical practice, tacrolimus dose adjustments are required based on the monitoring of tacrolimus trough blood concentrations [7]. As reported by the European Consensus Conference in 2009 [8], effective target trough tacrolimus concentrations should be positioned between 5 and 10 ng/mL, at least in the first year after transplantation.

Exposure below the recommended threshold increases the risk of transplant rejection and graft failure. Exposure above the recommended upper value, however, presents a risk of opportunistic infections (as a result of over-immunosuppression), malignancy, nephrotoxicity, tremors, diabetes, hypertension, and other adverse reactions.

The tacrolimus formulation LCPT (Life Cycle Pharma tacrolimus) is a once-daily extended-release tacrolimus formulation using MeltDose^®^ technology (Parma, Italy). This method allows the reduction of the drug particle size < 0.1 µm, resulting in better dissolution, drug release over the entire intestinal tract, better bioavailability, and lower intraday peak-to-trough fluctuation than other tacrolimus formulations [9,10]. Clinical trials provided evidence that de novo LCPT recipients achieved the target trough levels earlier, up to a 30% lower dose and with smaller dose adjustments compared to immediate-release (IR) tacrolimus [9,11].

There are few data on LCPT available on maintenance treatment regimens in de novo liver allograft recipients. The aim of this multicenter, non-interventional study was to assess, in real-life conditions, the tolerability and effectiveness of LCPT and its treatment schedules in hepatic allograft recipients.

## 2. Materials and Methods

This was a multicenter observational, non-interventional study (NIS) conducted in Austria and the Czech Republic to assess the tolerability and effectiveness of the extended-release tacrolimus MeltDose^®^ formulation LCPT (Envarsus^®^, Chiesi Farmaceutici S.p.A., Parma, Italy) in daily clinical practice. The data collection period was from July 2016 to August 2019.

A total of 8 clinical visits at baseline (i.e., LCPT initiation) on days 1, 7 ± 1, 14 ± 2, 21 ± 3, 60 ± 4, 90 ± 5, and 180 ± 7 (end of study visit) were scheduled. All timepoints allowed appropriate time windows to accommodate the integration of the study into the clinical routine. For the analysis, patients were assigned to visit numbers 1 to 8 according to their individual schedules. Tacrolimus blood trough levels were assessed at each study visit, and dosing was adjusted accordingly to maintain tacrolimus whole blood trough levels within the range of 5–20 ng/mL in the first 3 months after transplantation and in the range of 5–15 ng/mL afterwards. Physical examination, vital signs, laboratory tests, concomitant drugs, and documentation of adverse drug reactions and unrelated adverse events were assessed at each visit. An assessment of the probable relatedness of the reported events to immunosuppressive therapy was conducted post hoc. All procedures were performed according to the local clinical routine and no study-related procedures were mandated.

The duration of observation was 6 months and was established according to the European Medicines Agency’s guidelines on clinical investigation of immunosuppressants for solid organ transplantation [12]. Defining freedom from rejection as a reference for effectiveness; and risk of infection, risk of malignancies, or other known adverse effects of concomitant immunosuppressants (e.g., wound healing complications, nephrotoxicity etc.) as a marker of tolerability.

### 2.1. Eligibility Criteria

Adult (≥18 years old) recipients of liver transplants were included if they provided informed consent prior to any study-related documentation and agreed to participate in the study visits. Patients with known contraindications to LCPT, as per the summary of product characteristics (SmPC), and recent (≤30 days) participants in any other clinical trial or NIS were excluded.

### 2.2. Study Objectives

The primary objective was to assess the tolerability of LCPT. Secondary objectives included the determination of LCPT effectiveness; assessment of tacrolimus blood levels, LCPT dosing patterns, and dose adjustments; assessment of liver function using aspartate aminotransferase (AST), alanine aminotransferase (ALT), and gamma-glutamyl transferase (GGT); and monitoring of vital signs, such as systolic and diastolic blood pressure, pulse rate, weight, and body mass index (BMI). All outcome measures were documented from routine measurements as per the local standard.

### 2.3. Statistical Considerations

No study hypotheses were tested, and all statistical analyses were purely descriptive. For metric variables, the number of patients, mean, standard deviation (SD), median, minimum, and maximum were presented. For categorical variables, the numbers and percentages of patients are provided. Laboratory tests and vital signs were presented using descriptive statistics (n, mean, SD, median, range) for observed values and changes from baseline to each post-baseline visit, the number and percentage of patients with values above or below the normal value at each visit, and with shift tables showing the number and percentage of patients with values low/normal/high (relative to normal ranges) at baseline versus treatment visit. Time-to-event data were analyzed using the Kaplan–Meier method and summarized using medians, 95% confidence limits, and Kaplan–Meier survival plots. Sensitivity analysis was not performed. IBM SPSS Statistics (version 24; IBM Corp., Armonk, NY, USA) was used for statistical analysis.

The investigators were responsible for correctly documenting the data from patient records. The electronic case report form (eCRF) contained automatic validation checks and issued warnings during the documentation process if the entered values were out of the expected range (“plausibility checks”, e.g., age above 120). In addition, the eCRF showed warnings if essential data points were missing (e.g., the time of diagnosis). No monitoring or external documentation was conducted to comply with local data protection laws.

No formal sample-size calculations were performed. The sample size was determined based on feasibility assessments and past experiences from similar studies.

## 3. Guidelines

Reporting of this study followed the principles of the Reporting of Observational Studies in Epidemiology (STROBE) guidelines [13].

### Ethical Considerations

The study was conducted in compliance with the Declaration of Helsinki (1964 and amendments), the Declaration of Istanbul, current Good Clinical Practices, and all other applicable laws and regulations. The study was approved by the Institutional Ethics Committees (Medical University Graz No. 28-032; Vienna General Hospital No. 1298/2016) and registered in the Austrian National Study Registry (https://forms.ages.at/nis/listNis.do (accessed on 25 February 2016); study number: NIS005377). In the Czech Republic, the State Institute for Drug Control (SÚKL) was notified of the study (https://www.sukl.eu/modules/nps/index.php?h=study&a=detail&id=1241 (accessed on 25 February 2016); study identifier: 1609060000). No ethics committee approval was required by law at the time of this study (https://www.sukl.eu/sukl/ust-35-version-2 (accessed on 25 February 2016)). All participants provided written informed consent before participation.

## 4. Results

### 4.1. Baseline Characteristics

**Figure 1:**
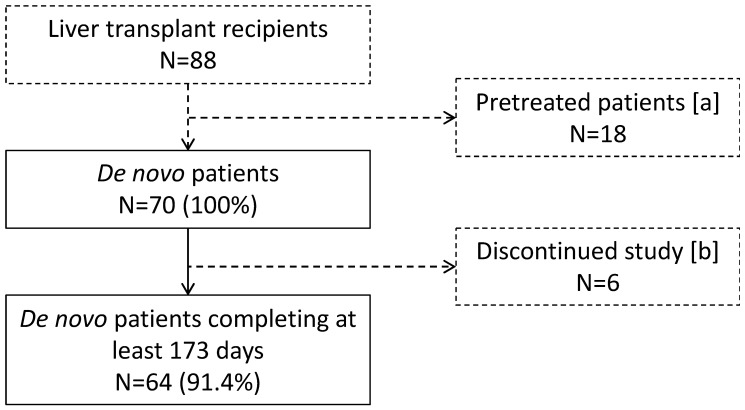
Patient disposition. [a] Switch from other tacrolimus (n = 14), unknown immunosuppressant (n = 2), or LCPT pretreatment (n = 2); no switch from prior cyclosporine. [b] As per the study protocol, eight visits over 180 ± 7 days were planned; however, only four visits were compulsory, and six patients were lost to follow-up.

Seventy de novo LCPT recipients following liver transplantation were analyzed and had at least one complete visit (60 patients from Austria, 10 from the Czech Republic); 91.4% (n = 64) completed eight visits (Figure 1). The patients were predominantly male (72.9%, n = 51). The mean (SD) age was 54 (11) years, and 85.7% (n = 60) were aged < 65 years old. The most frequently (five or more patients) documented underlying diseases were alcoholic cirrhosis (n = 38), hepatocellular carcinoma (n = 25), post-hepatitis cirrhosis (n = 10), sclerosing cholangitis (n = 9), and cryptogenic liver cirrhosis (n = 5); patients could have more than one relevant underlying liver disease. The patients with hepatocellular carcinoma (n = 25) presented as a second diagnosis of alcoholic cirrhosis (n = 12), posthepatitis cirrhosis (n = 6) non-alcoholic steatohepatitis (n = 2) and other types of cirrhosis (n = 5). Induction therapy was administered to 91.4% of patients (n = 64), of which 90.6% (n = 58/64) received antithymocyte globulin (ATG, at doses of 1.5 mg/kg bodyweight for 3 days), 9.4% (n = 6/64) received basiliximab at a dose of 20 mg on post-operative days (POD) 0 and 4. For patients without induction therapy or induction therapy with basiliximab, a daily dose of 2 g/day mycophenolate mofetil (MMF) was administered from POD 1. According to the center standards, an intravenous steroid bolus was administered intraoperatively and tapered to 10 mg prednisolone as maintenance therapy until post-operative week 12. After 3–6 months, patients without cholestatic or autoimmune liver disease were usually withdrawn from steroids. During the follow-up, no episode of acute cellular rejection was observed. Patient demographics, disease characteristics, and treatments are shown in Table 1.

### 4.2. LCPT Tolerability (Primary Objective)

The LCPT was well tolerated. Among the 70 de novo patients, 24 adverse events occurred in 8 patients, 21 were mild, 2 were moderate, and 1 was severe. Of these 24 adverse events, 17 were considered to be related to immunosuppressive therapy and 7 were considered unrelated. The most frequently occurring immunosuppressant-related events were renal failure (five events in three patients) and leukopenia (three events in three patients), but none of these were classified as serious events. Of the seven adverse events unrelated to immunosuppressive therapy, two were considered serious: one atrial flutter and one liver dysfunction occurring after transplantation of an extended criteria donor (ECD) liver (Table 2). No fatal events occurred. One discontinuation was reported for suspected LCPT-related worsening of pre-existing tremors of the hands; however, suspicion was not confirmed during the following investigation.

### 4.3. Tacrolimus Dosing and trough Levels over Time

**Figure 2:**
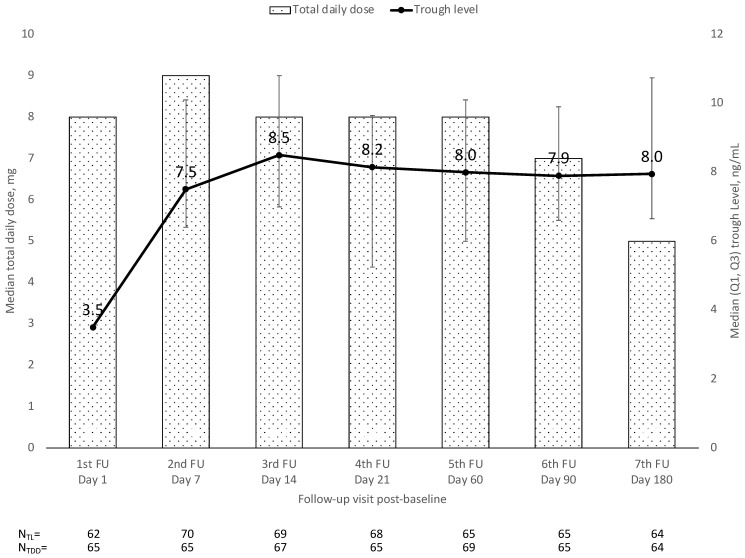
Tacrolimus trough levels and total tacrolimus daily dose over time. TDD = total daily dose; TL = trough level. The time points for each measurement are depicted as per the study protocol and are correlated with the median actual time points. For the analysis, patients were assigned to visit numbers 1 to 8 according to their individual schedules. Note: four patients received prograf (0.5 or 1 mg) during the first 7 days after transplantation because of the required intravenous drug administration during their ICU and/or intubation period before being switched to LCPT.

De novo patients were initiated on a median (Q1, Q3) dose of 8.0 (7.0, 8.0) mg per day, increasing to 9.0 (8.0, 10.0) mg after one week and subsequently decreasing to 5.0 (2.0, 6.0) mg at 6 months (Figure 2). The dose was decreased by a median (Q1, Q3) of 41.4% (−61.3%, −18.3%, *p* < 0.001) between initiation and 6 months (n = 60 with values at initiation and 6 months). The median (Q1, Q3) tacrolimus trough level increased steadily from 3.5 ng/mL (2.4, 6.1) at initiation to 8.5 ng/mL (5.6, 10.0) after two weeks and remained stable at approximately 8 ± 0.15 ng/mL thereafter (Figure 2). The median increase in trough level after stabilization (i.e., at the follow-up visit after a median of 7 days) did not change until the end of observation at month 6 (7.2% [−23.1%, 58.8%] median [Q1, Q3] increase, *p* = 0.361). The mean (SD) time to achieve tacrolimus target trough levels was 6.4 (4.6) days after a mean (SD) of 4.4 (4.0) dose adjustments.

### 4.4. Liver and Kidney Function over Time

**Figure 3:**
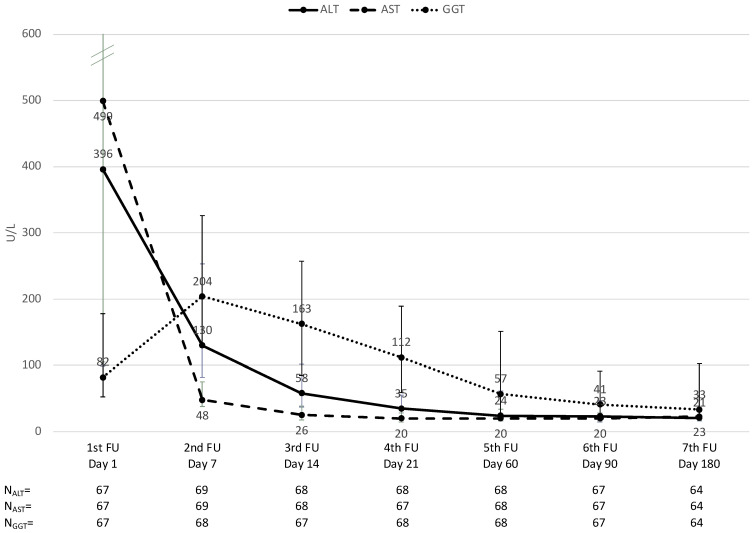
Liver function over time. AST, aspartate aminotransferase; ALT, alanine aminotransferase; GGT, gamma-glutamyl transferase. Note: the time points for each measurement are depicted as per the study protocol and correlate with the median actual time points. For the analysis, patients were assigned to visit numbers 1 to 8 according to their individual schedules.

**Figure 4:**
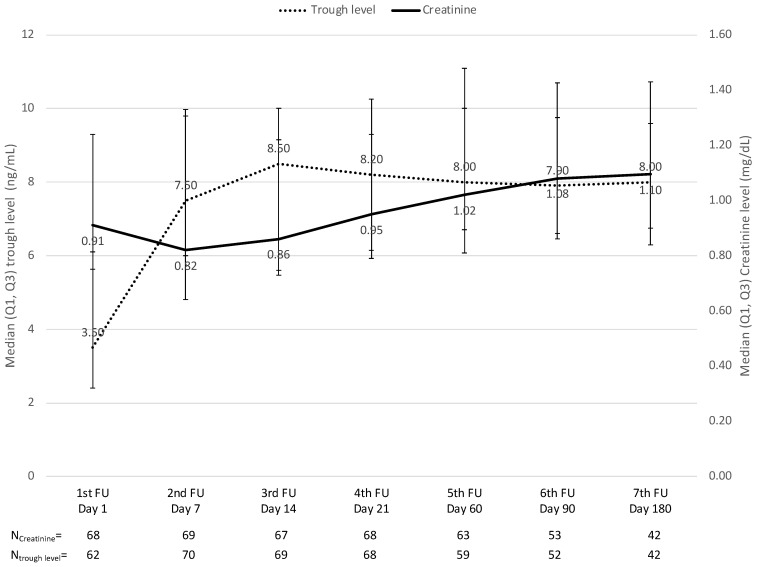
Serum creatinine and trough levels over time. Note: the time points for each measurement are depicted as per the study protocol and correlate with the median actual time points. For the analysis, patients were assigned to visit numbers 1 to 8 according to their individual schedules.

In de novo patients, liver function continuously improved, with the liver enzyme serum levels (ALT, AST, and GGT) continuously decreasing over time (Figure 3). Between day seven and 6 months, ALT decreased by a median (Q1, Q3) of 82.5% (−91.5%, −67.3%; *p* < 0.001, n = 63), AST by 56.3% (−71.4%, −26.5%, *p* < 0.001, n = 63), and GGT by 77.2% (−92.0%, −52.2%, *p* < 0.001, n = 62) but was already quite normal 2 weeks post-transplantation. Serum creatinine was not significantly increased by a median (Q1, Q3) of 31.6% (−10.6%, 67.8%, *p* = 0.085, n = 41) between day seven and 6 months and was not affected by the LCPT trough level (Figure 4).

## 5. Discussion

The present study investigated the tolerability and effectiveness of LCPT in de novo LCPT recipients following liver transplantation. In these patients, LCPT was well tolerated as there were no serious drug-related adverse reactions. Target trough levels were rapidly achieved within 14 days at a total daily dose of 8.0 mg after a median of 4.4 dose adjustments; the final dose at 6 months was 5.0 mg.

Tacrolimus is an important component of immunosuppression regimens in most liver transplant patients. However, it has a narrow therapeutic index that requires individualized dosing to achieve a satisfactory balance between maximum efficiency and tolerability. Complex inter- and intra-individual variability patterns in blood trough levels are considered to be due to low bioavailability and metabolic particularities. LCPT was developed to improve bioavailability and water solubility by creating a ‘solid solution’ of single tacrolimus molecules via a drug delivery technology called MeltDose^®^ [9]. This once-daily formulation allows for less peak-to-trough fluctuation and, consequently, better controllability of drug dosing [14]. Our study confirms the convenience of LCPT in daily clinical practice by showing a short titration period, a low number of dose adjustments, and a low maintenance dose.

The tolerability of LCPT was the primary outcome measure in the present study. However, adverse events have not been formally adjudicated for causal relationships with LCPT administration and have been reported as documented. However, the investigators classified the documented events as either probably related or unrelated to immunosuppression. Renal failure and hematological adverse events were most frequently observed. The observed adverse events corresponded to the known safety profile of LCPT [7] and no new safety signals were observed.

There are very few studies in the literature investigating liver transplant recipients newly initiated on LCPT [15,16], and our findings are in line with previous reports. The pharmacokinetics, safety, and efficacy of LCPT in de novo LCPT users with liver transplants were investigated in a phase 2 randomized study. In that study, once-daily LCPT was compared to the twice-daily immediate-release tacrolimus (IR-tac) formulation Prograf^®^ (County Kerry, Ireland) [15]. The pharmacokinetic profiles of these drugs were distinct, with a smaller degree of fluctuation with LCPT and a longer T_max_ compared to the IR-tac formulation. The increased bioavailability of LCPT enabled a similar systemic exposure at a lower dose. Approximately two-thirds of the patients in each group had therapeutic trough levels after 14 days, albeit with fewer dose adjustments in the LCPT group (3.9/patient versus 4.8/patient). A single-center, retrospective study compared LCPT with the once-daily prolonged-release tacrolimus (PR-tac) formulation Advagraf^®^ during the first 30 days after transplant [16]. LCPT resulted in faster attainment of therapeutic trough levels than comparable doses of the PR-tac formulation; after stabilization, the maintenance LCPT dose was 25% than the PR-tac formulation [16]. The mean LCPT maintenance dose at post-operative day 15 and day 30 was 5 mg/day. A possible explanation for the rapid attainment of target trough levels may be the main pathway of LCPT resorption through the large intestine [17]. This also allows greater independence from gut motility in the post-operative phase.

The fact that LCPT provides stable tacrolimus levels even in the early postoperative might contribute to the fact that there was no acute rejection observed during follow. This is in line with a previous study that reported a rejection rate of around 1% [18].

The present study has several strengths and limitations. The completeness of data with very few patients lost to follow-up is a strength and is due to the fact that follow-up care was provided at the transplant center. However, limitations typically inherent to observational study designs need to be considered. This study was observational and did not include a control group. As previously mentioned, LCPT tolerability as the primary outcome measure was not adjudicated for causality, possibly leading to an overestimation of actual adverse drug reactions. On the other hand, underreporting of adverse events is common in the setting of observational studies [19]. Overall, phase 3 trials found a similar safety profile for LCPT compared to other tacrolimus formulations, as extensively reviewed by Grinyó et al. [9] The present study did not mandate any study-related visits at pre-specified time points but rather followed the physician-defined individual patients’ schedules. The analysis of the actual median time point of each visit, however, correlated well with the planned study schedule.

In summary, most studies have focused on stable patients converting to LCPT from other tacrolimus formulations. After LCPT initiation, tacrolimus target trough levels were rapidly achieved in the studied de novo LCPT users receiving liver transplants, while the initial total daily tacrolimus was reduced after day 60 with few dose adaptations. Liver function rapidly improved, whereas kidney function remained normal. LCPT was well-tolerated in this population. This study fills an evidence gap regarding liver transplant patients newly initiating LCPT and shows that LCPT administered once daily may be an attractive alternative to other tacrolimus formulations in liver transplant recipients.

## Figures and Tables

**Table 1:** Patient demographics and disease characteristics of de novo LCPT liver transplant patients at baseline.

	Liver Transplant Patients De Novo, n = 70
Male sex, n (%)	51 (72.9)
Mean (SD) age, years	54 (11.2)
Age group, n (%)	
<65 years	60 (85.7)
≥65 years	10 (14.3)
Mean (SD) weight, kg	78 (12.9)
Mean (SD) height, cm	171 (11.9)
Mean (SD) BMI, kg/m^2^	27 (5.8)
Lab MELD (SD)	17 (7.2)
Underlying disease, n [a]	
Alcoholic cirrhosis	38
Hepatocellular carcinoma	25
Post-hepatitic c cirrhosis	10
Sclerosing cholangitis	9
Cryptogenic liver cirrhosis	5
Acute hepatic failure	4
Cirrhosis of unknown causes	4
Wilson disease	3
Non-alcoholic steatohepatitis	3
Primary biliary cirrhosis	3
Secondary liver tumors	3
Autoimmune cirrhosis	2
Others	5
Immunosuppressive regimen [b, c], n (%)	
Induction therapy	64/70 (91)
ATG	58/64 (90.6)
Basiliximab	6/64 (9.4)
Steroids	70 (100)
MMF	12 (17.1)

ATG, anti-thymocyte globulin; BMI, body mass index; CNI, calcineurin inhibitor; LCPT, extended-release tacrolimus MeltDose^®^ formulation; MMF, mycophenolate mofetil; SD, standard deviation. [a] A given patient could have more than one underlying disease; therefore, only the number of patients with a given underlying disease was provided. [b] All patients received LCPT after baseline and had no prior use of any immunosuppressants, that is, de novo LCPT users. [c] As reported at the first follow-up visit on the day of transplantation, that is median day 0.0. [d] Four patients received prograf (0.5 or 1 mg) during the first 7 days after transplantation because of the required intravenous drug administration during their ICU and/or intubation period before being switched to LCPT.

**Table 2:** Safety events by MedDRA terms.

Number of Events	Adverse Events (n = 70)
Any AE	**24**
Mild AE	21
Moderate AE	2
Severe AE	1
ADRs (related to immunosuppression)	**17**
Leukopenia NOS	3
Arterial hypertension	2
Renal failure	5
Abdominal infection	1
Urinary tract infection	2
CMV infection	2
Epilepsy NOS	1
Hyperglycemia NOS	1
AEs (unrelated to immunosuppression)	**7**
Hemorrhagic anemia	2
Atrial flutter *	1
Hepatic enzyme increased	1
Hepatic function abnormal *	1
Collapse circulatory	1
Insomnia	1

n (all patients) = 70, n (male patients) = 51, n (female patients) = 19; AE, adverse event (unrelated to immunosuppression); ADR, adverse drug reaction (related to immunosuppression); CMV, cytomegalovirus; NOS, not otherwise specified; * Serious unrelated adverse event; both patients did not discontinue LCPT due to serious adverse events. Bold data are the total number in each categorie.

## Data Availability

Chiesi Pharmaceuticals GmbH owns the data, and all authors have access to it. Qualified researchers may request data from the corresponding authors.

## Abbreviations

ADR	adverse drug reaction
AE	adverse event
ALT	alanine aminotransferase
AST	aspartate aminotransferase
BMI	body mass index
CNI	calcineurin inhibitor
ECD	extended criteria donor
eCRF	electronic case report form
FK506	tacrolimus
GGT	gamma-glutamyl transferase
IR	immediate-release
LCP	Life Cycle Pharma
LCPT	LCP-tacrolimus
mAb	monoclonal antibody
MMF	mycophenolate mofetil
NIS	non-interventional study
NOS	not otherwise specified
POD	post-operative day
Q1	25% quartile
Q3	75% quartile
SD	standard deviation
SADR	serious adverse drug reaction
SAE	serious adverse event
SmPC	summary of product characteristics
STROBE	Reporting of Observational Studies in Epidemiology
SÚKL	Czech State Institute for Drug Control
